# Resection of the Knee Joint for Tuberculosis

**Published:** 1890-12

**Authors:** 


### RESECTION OF THE KNEE JOINT FOR TUBERCULOSIS.

Dr. Schluter reports one hundred cases of tuberculosis of the knee joint occurring in persons who had passed the age of twenty, in which resection was performed. The operation usually performed was that of Volkmann, in which the patella is sawed through transversely; but sometimes the joint was exposed by a curved incision below the patella. All the diseased tissues were carefully removed and the synovial membrance extirpated; the bones as a rule were not sutured. Healing usually took place without complications, the average period being two months; in some of the cases recovery was retarded by suppuration and recurrences of the tuberculosis The results in the 100 cases reported, show that 44 patients were completely cured, 3 not improved, 11 underwent amputations, 32 died, and 10 were lost sight of. In the 44 cases where a cure was effected, recovery was still permanent ten years after the operation. The author has also collected from the literature 187 cases of resection of the knee for tuberculosis in adults, with 30 per cent of cures. His conclusion is that resection in persons in advanced life gives a useful limb in 64 per cent. of those operated upon and within half a year after the operation.—*Deutsche Zeitsshrift f. Chirurgie.*

